# Effect of Medical Fee Schedule Revisions on the Expansion of Nutritional Support Teams in Japan: An Interrupted Time-series Analysis

**DOI:** 10.31662/jmaj.2022-0207

**Published:** 2023-06-12

**Authors:** Sadaharu Asami, Kevin Y Urayama, Daiki Kobayashi, Kohei Onozaki, Osamu Takahashi

**Affiliations:** 1 Department of Cardiology, Musashino Tokushukai Hospital, Tokyo, Japan; 2 Division of Epidemiology, Graduate School of Public Health, St. Luke’s International University, Tokyo, Japan; 3 Department of Social Medicine, National Center for Child Health and Development, Tokyo, Japan; 4 Division of General Internal Medicine, Department of Internal Medicine, Tokyo Medical University Ibaraki Medical Center, Ibaraki, Japan; 5 Fujita Health University, Aichi, Japan; 6 Division of Health Policy and Management, Graduate School of Public Health, St. Luke’s International University, Tokyo, Japan; 7 Department of Internal Medicine, St. Luke’s International Hospital, St. Luke’s International University, Tokyo, Japan

**Keywords:** Interrupted time-series analysis, Nutrition support team, Health policy, Fee schedule

## Abstract

**Introduction:**

In 2018, the fee schedule for nutrition support teams (NSTs) in Japanese hospitals was changed. The change was intended to encourage more hospitals to establish NSTs, and this study aims to investigate whether this change had the desired effect. Specifically, we will look at the proportion of hospitals with NSTs before and after the 2018 revision to see if there was a significant increase in the number of hospitals with NSTs.

**Methods:**

The study analyzed administrative data from 10 Japanese prefectures dating from June 2015 to September 2021 using an interrupted time-series design. The analysis focused on all acute care hospitals within these prefectures and measured the percentage of hospitals with NSTs. Subgroup analyses were conducted based on hospital size and functions. In April 2018, the intervention, a fee schedule revision, was implemented.

**Results:**

We analyzed 1,471 acute care hospitals. Immediately after the intervention, the percentage of hospitals with NSTs increased by 4.59% (95% CI = 3.92%, 5.26%) and by 0.66% (95% CI = 0.57%, 0.75%) quarterly thereafter. We observed a marked increase in NST formation among large-sized (20.9%), medium-sized (28.0%), and highly acute care hospitals (hospitals with emergency medical care centers and intensive care units, 22.3% and 23.6%, respectively). We also noted a moderate increase among hospitals with convalescent rehabilitation units (10.1%) and a modest increase among small-sized hospitals (6.9%).

**Conclusions:**

Relaxation of the NST fee requirement increased the proportion of hospitals with NSTs in Japan, especially among larger and highly acute care hospitals.

## Introduction

An international survey has shown that up to 40% of inpatients suffer from disease-related malnutrition ^(1)^, leading to higher mortality, extended hospitalization, and increased readmissions ^(2)^. However, adequate nutritional support can alleviate these adverse outcomes in malnourished inpatients. Based on the results of a systematic review of randomized control trials, nutritional support for medical inpatients may reduce mortality ^(3)^. A nutrition support team (NST) is a multidisciplinary team composed of at least a doctor, nurse, dietician, and pharmacist with specialized skills for nutritional support. NSTs are dedicated to supporting the nutritional needs of patients admitted to hospitals. Many international guidelines recommend that acute care hospitals establish NSTs, as they have been associated with improved patient outcomes ^(4), (5), (6)^.

The Japanese healthcare system’s foundation is fee-for-service reimbursement per the national fee schedule ^(7)^. To obtain reimbursement from the Claims Review and Reimbursement Organizations for the remaining 70%-90% of medical fees, all medical facilities in Japan must follow the calculation requirements for medical services set by the Ministry of Health, Labor, and Welfare (MHLW) ^(8)^. The fee schedule undergoes revisions every two years to manage overall expenditures and financially incentivize healthcare providers to alter their practices ^(9)^.

Until recently, there were no financial incentives for NST activities in Japan, and the fee for NST services was hoped to be included in the medical fee schedule. To facilitate the establishment of NSTs, the MHLW introduced the NST fee under the medical fee schedule in 2010 ^(10)^. After certification, hospitals were eligible to claim the NST fee per service, and such a system was expected to promote the formation of NSTs in acute care hospitals.

However, the intricate requirement for hospital certification was an obstacle for many hospitals: one out of four qualified NST members should have been exclusively engaged in NST operations ^(11)^. Consequently, in 2014, less than 22% of all acute hospitals in Japan were certified by the MHLW as having NSTs ^(12)^. In 2015, research funded by the MHLW confirmed that the calculation required for the NST fee was stringent, and relaxation of these requirements was needed to promote NST activity ^(13)^. In response, the MHLW relaxed the requirement for NST certification in the 2018 fee schedule revision, and hospitals were no longer required to employ a member exclusive to the NST ^(14)^.

The revisions were expected to expand NST formation in hospitals across Japan. However, reports have yet to assess the effects of this policy revision and the resultant changes in NST resources provided by hospitals to the Japanese population. In this large-scale nationwide examination of NST services provided among acute care hospitals, we aimed to investigate whether this relaxation increased the proportion of hospitals with NSTs.

## Materials and Methods

### Data source

The data used in this study were obtained from the Prefectural Bureau of Health and Welfare (PBHW). We obtained a list of all hospitals certified under the medical fee system, which is renewed monthly and includes hospital characteristics, such as hospital type, number of beds, and various hospital functions. We targeted Tokyo and the 9 surrounding prefectures, and obtained quarterly lists from June 2015 to September 2021 ^(15)^. We selected this area because it is Japan’s central area and consists of densely and sparsely populated prefectures.

### Population

Acute care hospitals served as the unit of evaluation for this study. This study’s definition of acute care hospitals includes only those with 20 or more general beds. This definition was chosen to exclude long-term care hospitals from the study’s population. We extracted data on the included hospitals from the lists obtained from the PBHW. Alongside the number of beds, the extracted data included whether the hospitals had the following: emergency medical care center (EMCC), intensive care unit (ICU), convalescent rehabilitation unit (CRU), and NST. We selected these characteristics because national guidelines recently stated the need for nutritional support for critically ill patients and patients during rehabilitation ^(16), (17)^.

### Ethical approval

The Institutional Review Board of St. Luke’s International University reviewed and approved this study protocol, approval number 21-R129. The board confirmed no concerns about consent-to-participate issues in this study because it used only open-access data.

### Outcome measure

We measured the outcome as the percentage of hospitals that reporting NSTs during each quarter over the entire study period. For the percentage, the numerator was the number of hospitals with NSTs, and the denominator was all the hospitals included during that quarter.

### Statistical analysis

We performed an interrupted time-series analysis (ITSA) to estimate changes in levels and trends in the percentage of hospitals with NSTs after the intervention of the 2018 fee schedule revision ^(18)^. We defined the pre-intervention period as May 2015 to March 2018, the time of intervention as April 2018, and the post-intervention period as May 2018 to September 2021. According to government research, the main reason for not claiming the fee for NST services was the difficulty in securing qualified members ^(19)^. In Japanese hospitals, the number of staff per 100 beds tends to decrease as the total number of hospital beds decreases ^(20)^. Therefore, we examined whether the effect of this revision differed according to hospital size. The acute care hospitals were categorized into three size groups based on their number of general beds: small, medium, and large with fewer than 200, between 200 and 399, and 400 or more beds, respectively. We also analyzed the percentage of hospitals with NSTs among hospitals in specific subgroups that were defined as having the following functions: EMCC, ICU, and CRU. Some hospitals with CRUs had no general beds and belonged to long-term care hospitals; therefore, we decided to include long-term care hospitals in our samples only when calculating the percentage of hospitals with CRUs.

We searched for other medical fee schedule revisions that could have affected the outcome, but none were identified. We took autocorrelation into account using regression with the Newey-West standard errors with one lag ^(21)^. Thereafter, we conducted the Cumby-Huizinga test to ensure that our model could account for the autocorrelation structure ^(22)^. We excluded seasonality because our data’s scatter plot did not show seasonality’s influence. All statistical analyses were performed using Stata 16.1 (StataCorp).

## Results

**Figure 1.**
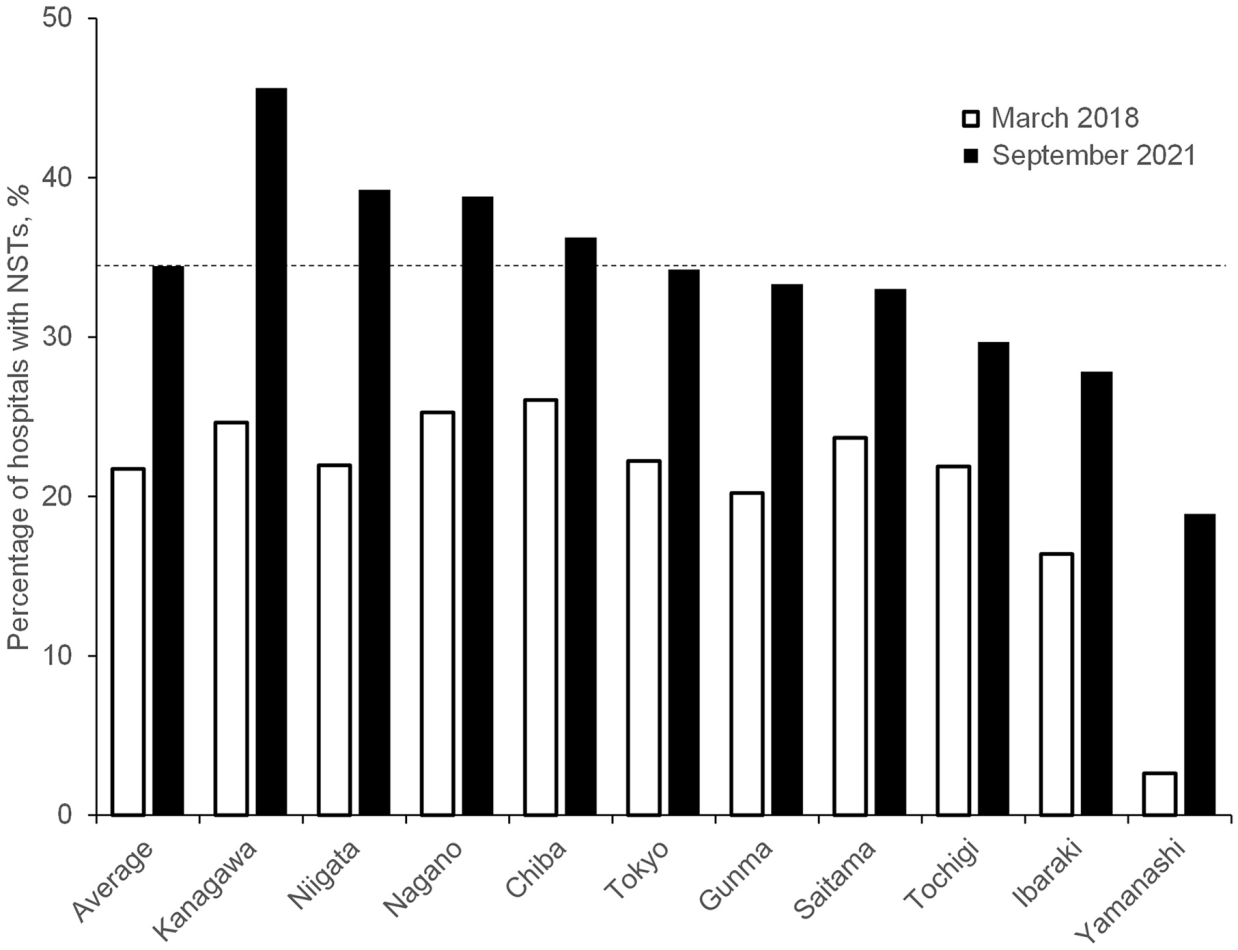
Percentage of hospitals with Nutrition Support Teams (NSTs) in the 10 Japanese prefectures in March 2018 and September 2021. “Average” means the average of the 10 prefectures.

As of September 2021, the characteristics of the sampled hospitals were similar to those of all acute care hospitals in Japan, as shown in Table 1. The study included 30% (1,471/4,822) of the total number of acute care hospitals in Japan. Looking at the data by prefecture, there were apparent differences in the percentage of hospitals with NSTs within the 10 prefectures at the start and end of the study period, as shown in Figure 1.

**Table 1.** Comparison of the Characteristics of Acute Care Hospitals between This Study and All Hospitals in Japan.

	Tokyo and nine surrounding prefectures	All 47 prefectures
No. of acute care hospitals in this area	1471	4822
No. of general beds	*n* (%)	*n* (%)
<200 (small-sized)	1042 (70.8)	3507 (72.7)
200-399 (medium-sized)	247 (16.8)	797 (16.5)
≥400 (large-sized)	182 (12.4)	518 (10.7)
subgroups	*n* (%)	*n* (%)
Hospitals with EMCC	96 (6.5)	276 (5.7)
Hospitals with ICU	229 (15.6)	685 (14.2)
Hospitals with NST	507 (34.5)	1673 (34.7)

NST, nutrition support team; EMCC, emergency medical care center; ICU, intensive care unit.

### Main analysis

The percentage of hospitals with NSTs increased by 4.59% (95% CI = 3.92%, 5.26%) immediately after the intervention and by 0.66% (95% CI = 0.57%, 0.75%) quarterly thereafter, as shown in Table 2. The Cumby-Huizinga test revealed that autocorrelation existed at lag 1, but was absent at any other higher lag orders. Therefore, the assumed model accounted for this autocorrelation.

**Table 2.** Interrupted Time-Series Analysis of the Percentages of Hospitals with NSTs.

Absolute change in percentage		
(No. of hospitals with NSTs by quarter/No. of all hospitals by quarter) × 100, *%*		
	Level change immediately after intervention (95% CI)	Quarterly trend after intervention (95% CI)
All acute care hospitals	4.59 (3.92-5.26)***	0.66 (0.57-0.75)***
No. of general beds
<200 (small-sized)	1.71 (0.82-2.59)***	0.41 (0.32-0.50)***
200-399 (medium-sized)	5.70 (0.60-10.80)*	1.62 (1.09-2.15)***
≥400 (large-sized)	7.66 (3.21-12.12)**	1.26 (0.77-1.75)***
Subgroups
Hospitals with EMCC	7.21 (2.42-12.00)**	1.09 (0.59-1.60)***
Hospitals with ICU	7.74 (2.39-13.10)**	1.30 (0.71-1.90)***
Hospitals with CRU	3.42 (0.83-6.02)*	0.50 (0.23-0.77)***

NST, nutrition support team; CI, confidence interval; EMCC, emergency medical care center; ICU, intensive care unit; CRU, convalescent rehabilitation unit.* p* < 0.05, 0.01, 0.001 are indicated by *, **, ***.

### Subgroup analysis

**Figure 2.**
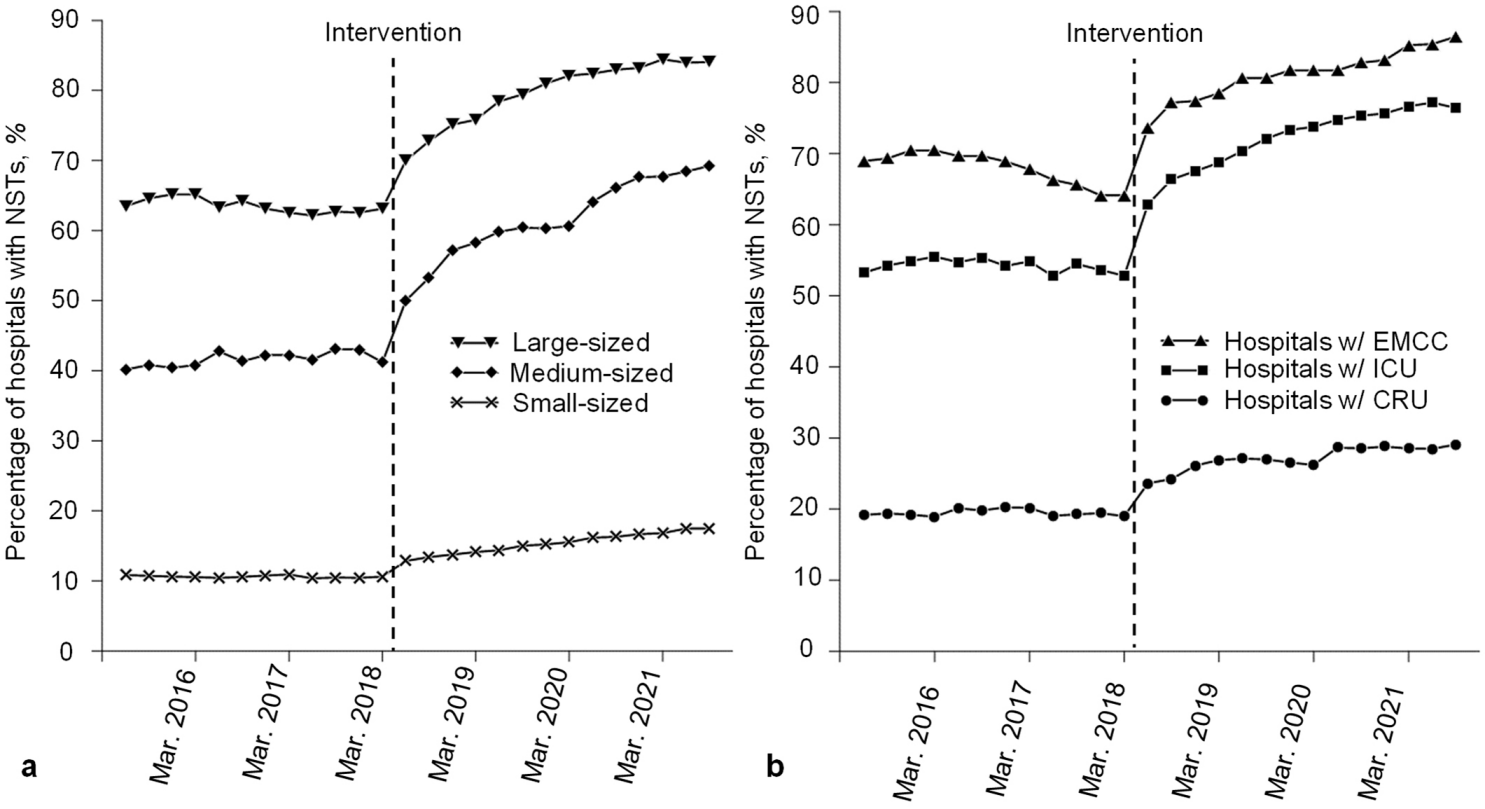
a. Quarterly trends in the percentages of hospitals with Nutrition Support Teams (NSTs) among hospitals of three sizes. The intervention began in April 2018. b. Quarterly trends in the percentages of hospitals with Nutrition Support Teams (NSTs) among hospitals in specific subgroups. Each dot shows the actual number of hospitals with NSTs among each group. The intervention began in April 2018. EMCC, emergency medical care center; ICU, intensive care unit; CRU, convalescent rehabilitation unit.

Table 2 shows that even after stratifying hospitals by size, marked increases in NSTs for each group size were observed based on both immediate level and trend changes. The effect of the intervention differed greatly by hospital size for both the immediate level and the trend changes regarding the percentage of medium- and large-sized hospitals. These showed a marked increase compared to those of small-size hospitals. As shown in Figure 2, at the endpoint of this study, the percentage of hospitals with NSTs differed considerably between the large-, medium-, and small-sized hospitals (84.1% in the large, 69.2% in the medium, and 17.5% in the small-size hospitals).

Subgroup analysis revealed that both the levels and trends increased significantly among all groups, as shown in Table 2. However, the effect of the intervention differed greatly by hospital function, as shown in Figure 2. Among hospitals with EMCCs and ICUs, which are regarded as highly acute care hospitals, steep rising trends in the NSTs’ percentage were observed, similar to those of medium- and large-sized hospitals. By the end of the study period, the percentage of hospitals with NSTs among hospitals with EMCCs and ICUs reached 86.4% and 76.4%, respectively. Comparing these subgroups, the increasing trend in the percentage of NSTs among hospitals with CRUs was moderate. However, care should be taken in interpretation since the sample of hospitals with CRUs includes long-term care hospitals.

## Discussion

This study revealed that the relaxed calculation requirement for NST fees in 2018 increased the proportion of hospitals with NSTs in Japan. Increases were observed immediately after the intervention, followed by a consistently increasing trend during the study period. The intervention increased NSTs among all types of hospitals, but the degree of increase over the entire post-intervention period appeared different. A marked increase was observed among large-sized, medium-sized, and highly acute care hospitals (i.e., hospitals with EMCCs and those with ICUs). However, only a moderate increase was observed among small-sized hospitals and hospitals with CRUs. Some of these findings are consistent with a previous small survey conducted by the MHLW, showing that the prevalence of hospitals with NSTs was higher in facilities with more beds than in those with fewer beds ^(19)^.

To the best of our knowledge, this study pioneers the evaluation of the effect of a medical fee schedule revision on hospital behavior using ITSA. An ITSA is considered one of the strongest quasi-experimental designs and has been used to assess the effect of payment interventions in the healthcare field ^(23), (24)^. This study also revealed the current prevalence of acute care hospitals with NSTs in Japan. To date, limited reports exist regarding the prevalence of NSTs in acute hospitals worldwide, except for a survey conducted in the United Kingdom ^(25)^. Furthermore, the data in our study were reliable as they were obtained from administrative sources. In the Japanese medical fee system, every institution must register the number of beds and the functions performed with the PBHW for acceptance to receive payments for insurance-covered treatment ^(8)^. Hence, we consider our data high-quality for all hospitals that provide insurance-covered treatment.

Although the number of hospitals with NSTs has increased due to financial incentives, it seems unlikely that the current prevalence of acute care hospitals with NSTs in Japan is sufficient. NST prevalence for acute care hospitals in Japan with ≥100 general beds was 55%, which was substantially lower than the United Kingdom’s prevalence of 80% ^(25)^. Thus, NST activities can be conducted even in hospitals that do not claim an NST fee. However, using the NST fee as a financial resource for maintaining and stably developing team medicine is preferable, especially in private hospitals ^(13)^. Hence, further exploration of the low prevalence of hospitals that claim NST fees is required to encourage more NSTs throughout Japan.

Based on the MHLW’s questionnaire survey in 2018, there were two main reasons for not claiming the fee for NST service. Firstly, there was minimal benefit due to the calculation requirement for the fee and setting up the NST team. Secondly, there was difficulty in securing qualified members ^(19)^. One potential solution to the first issue includes providing additional financial incentives, such as relaxation of the calculation requirement, raising the fee, or changing the payment method for the NST service from “fee-for-service” to “per admission.” However, these solutions may lead to a decrease in the quality of NST activity and an inappropriate increase in costs due to the excessive provision of services ^(26)^. Therefore, outcome data must be gathered, and approaches for financial incentives should be critically discussed using evidence-based policymaking. Regarding the difficulty in securing qualified members, becoming a qualified member requires the completion of a specific training course offered by the relevant medical society or dietitian association. If access to this training is not readily available, it would be challenging for hospitals to claim the NST fee. Therefore, the accessibility of this education should be studied and addressed. This study would identify the reasons for the difference in the percentage of hospitals with NSTs within the 10 prefectures analyzed in this study.

Our study had some limitations. Firstly, the number of hospitals with NSTs in this study was likely underestimated since the count was based solely on administrative data. This is because some hospitals operate NSTs without certification by the PBHW, meaning there are likely more NSTs in Japan than are officially recognized. However, our study specifically focused on hospitals in Japan that had been certified by the MHLW as having NSTs. We excluded hospitals that may have been certified by other organizations or societies with NSTs, so our scope was limited to MHLW-certified hospitals only. Secondly, there may have been other changes that occurred around the same time as the intervention (a fee schedule revision in April 2018) that could have affected the study outcome beyond the revision. Although we confirmed that there were no other fee schedule revisions during the study period that could have promoted NST formations, there may have been other time-varying confounders that were not adjusted. Thirdly, while we demonstrated an increase in the number of NSTs, we could not determine whether the NST activities in each hospital had also increased. There may have been hospitals that were only certified to have NSTs but did not provide NST services. Although we could not determine the status of activities at individual hospitals, we confirmed an increase in the overall activity of NSTs through the open National Database by checking the number of claims for the NST fee ^(27)^.

In conclusion, the relaxation of the calculation required for the NST fee increased the proportion of hospitals with NSTs in Japan, especially among larger and highly acute care hospitals. To further disseminate NSTs, financial incentives and various measures are needed to secure qualified NST members.

## Article Information

### Conflicts of Interest

None

### Acknowledgement

We would like to thank Editage [http://www.editage.com] and David Wright for editing and reviewing this manuscript regarding the English language.

### Author Contributions

Sadaharu Asami conceived and designed the study; Sadaharu Asami and Daiki Kobayashi analyzed the data; Sadaharu Asami wrote the manuscript; Kevin Urayama revised the manuscript; and Kohei Onozaki and Osamu Takahashi supervised the study. All authors critically revised the report, commented on manuscript drafts, and approved the final report.

### Approval by Institutional Review Board (IRB)

The Institutional Review Board of St. Luke’s International University reviewed and approved this study protocol, approval number 21-R129. The board confirmed that there is no concern about the consent-to-participate issue in this study, as this study used only open-access data.
